# Supplementary figures and images for: Mechanical Properties of Growing Melanocytic Nevi and the Progression to Melanoma
Source: PLoS One. 2014 Apr 7;9(4):e94229. doi: 10.1371/journal.pone.0094229 (PMC3978068; doi:10.1371/journal.pone.0094229)

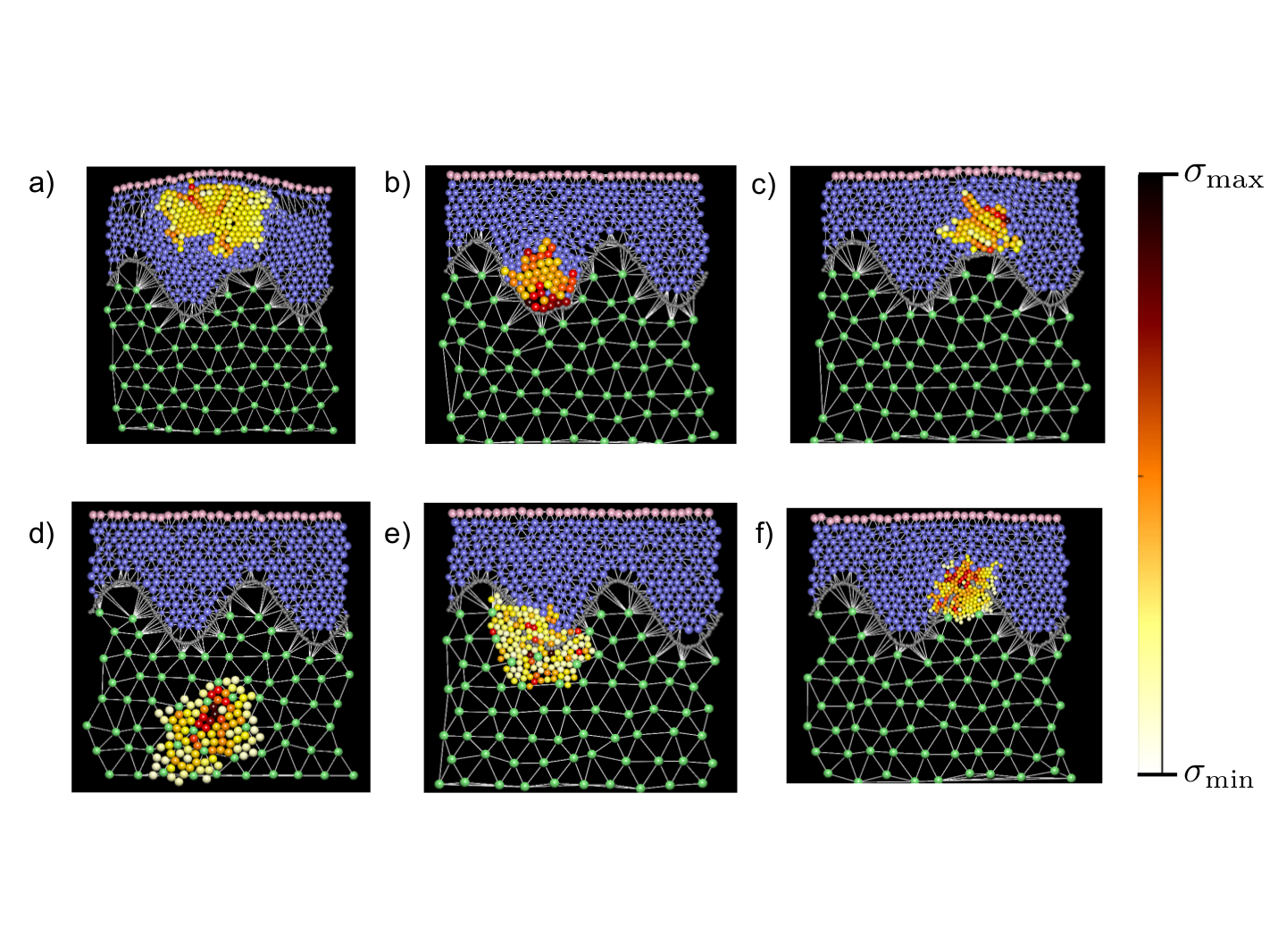

Supplement: Figure S1 — Morphology of nevi for different locations of the initiating cell for pressure dependent growth. Illustration of the results of numerical simulations for nevi grown from melanocytes located in different positions in the skin and for different mechanical properties of the basal membrane. The conditions are the same as in Fig. 5, but here the growth depends on the compressive stress acting on each cell. Growing melanocytes are shown with a varying color that reflects their compressive stresses according to the color bar. The maximum and minimum for each configuration are equal to (in kPa): a) 7.46 , b) , c) d) e) f) . (TIFF) [file pone.0094229.s001.tiff]

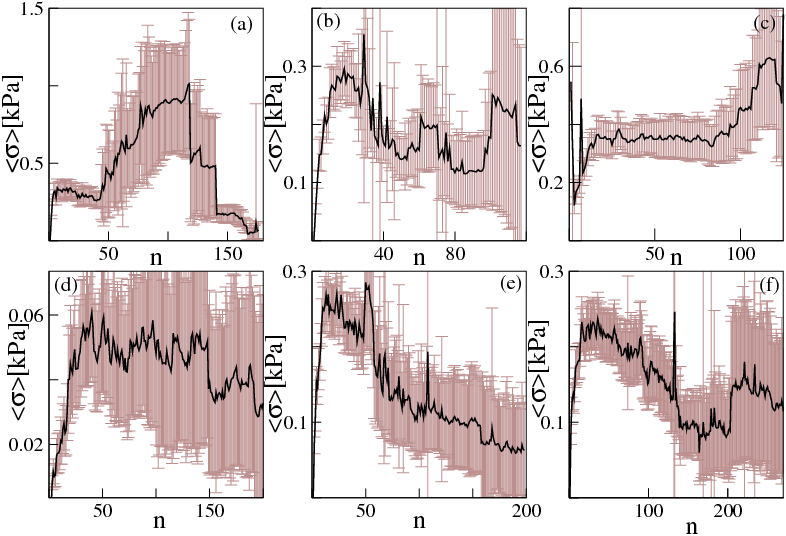

Supplement: Figure S2 — Mechanical stresses in pressure dependent growth of nevi. We report the evolution of the average compressive stress experienced by the melanocytes composing the nevus as a function of the size of the nevus, quantified by the number of cells n, for the same conditions as in Fig. S1. The error bars represent the standard error of the mean. The different panels represent different initial locations: a) in the middle of the epidermis, b) in the minima of a strong basal membrane, c) in the maxima of a strong basal membrane, d) in the dermis, e) in the minima of a weak basal membrane, f) in the maxima of a weak basal membrane. (TIFF) [file pone.0094229.s002.tiff]
